# Predicting VO_2peak_ from Submaximal- and Peak Exercise Models: The HUNT 3 Fitness Study, Norway

**DOI:** 10.1371/journal.pone.0144873

**Published:** 2016-01-21

**Authors:** Henrik Loe, Bjarne M. Nes, Ulrik Wisløff

**Affiliations:** 1 K.G. Jebsen Center of Exercise in Medicine at Department of Circulation and Medical Imaging, Norwegian University of Science and Technology, Trondheim, Norway; 2 Valnesfjord Rehabilitation Center, Valnesfjord, Norway; University of Rome, ITALY

## Abstract

**Purpose:**

Peak oxygen uptake (VO_2peak_) is seldom assessed in health care settings although being inversely linked to cardiovascular risk and all-cause mortality. The aim of this study was to develop VO_2peak_ prediction models for men and women based on directly measured VO_2peak_ from a large healthy population

**Methods:**

VO_2peak_ prediction models based on submaximal- and peak performance treadmill work were derived from multiple regression analysis. 4637 healthy men and women aged 20–90 years were included. Data splitting was used to generate validation and cross-validation samples.

**Results:**

The accuracy for the peak performance models were 10.5% (SEE = 4.63 mL⋅kg^-1^⋅min^-1^) and 11.5% (SEE = 4.11 mL⋅kg^-1^⋅min^-1^) for men and women, respectively, with 75% and 72% of the variance explained. For the submaximal performance models accuracy were 14.1% (SEE = 6.24 mL⋅kg^-1^⋅min^-1^) and 14.4% (SEE = 5.17 mL⋅kg^-1^⋅min^-1^) for men and women, respectively, with 55% and 56% of the variance explained. The validation and cross-validation samples displayed SEE and variance explained in agreement with the total sample. Cross-classification between measured and predicted VO_2peak_ accurately classified 91% of the participants within the correct or nearest quintile of measured VO_2peak_.

**Conclusion:**

Judicious use of the exercise prediction models presented in this study offers valuable information in providing a fairly accurate assessment of VO_2peak_, which may be beneficial for risk stratification in health care settings.

## Introduction

Peak oxygen uptake (VO_2peak_) is widely referred to as cardiorespiratory fitness (CRF) [1], and is inversely linked to cardiovascular disease, hypertension, certain cancers, metabolic syndrome [2,3], and all-cause mortality [4]. At present there is no consensus identifying a precise threshold of cardiorespiratory fitness to be associated with increased cardiovascular risks. However, values below 8 METs and 6 METSs in healthy men and women, respectively, are linked with higher all-cause mortality and adverse cardiovascular effects [4]. Additionally, data suggest that MET levels > 9 and > 7 (vs. lower MET levels) among men and women, respectively, is associated with a mortality risk reduction of ≥ 50% over an average 8 years follow-up [5]. Despite being an essential health indicator, VO_2peak_ is rarely assessed in health care settings [5,6], likely because direct gas analysis measurements of VO_2peak_ is expensive, necessitate the use of advanced equipment, and trained personnel [2]. However, reliable and valid prediction models should be considered as several studies have shown that either directly measured or estimated VO_2peak_ enhance CVD-mortality prediction beyond traditional risk factors [7,8].

Although a maximal test is considered a safe practice, complications and adverse effects occur, normally linked to underlying disease [9]. Consequently, health care personnel should monitor when testing individuals at high risk.

There exist several VO_2peak_ prediction models in the literature. Common limitations in these models are the use of uniform age samples [10–12], only one-gender represented [13–17], as well as models being based on subjects with homogenous cardiorespiratory fitness levels [10,12,13,15,18]. Hence, they yield fair VO_2peak_ predictions only in subjects similar to those used in generating the model [2,19].

Therefore, the aim of the present study was to develop VO_2peak_ prediction models from both submaximal- and peak treadmill performance, on the basis of data from a large healthy population of both men and women 20–90 years, with a great diversity in measured VO_2peak_. If these models show fair predictive accuracy they will provide a safe and feasible method for estimating VO_2peak_ for a wide variety of people.

## Methods

### Study sample

In 2006–2008, the total population above 20 years of age in Nord-Trøndelag county, in Norway, were invited to the third wave of the HUNT study (HUNT 3). Out of a total population of 94194, 54% accepted the invitation (n = 50821). A sub-study (The HUNT Fitness Study) invited healthy subjects (without cardiovascular disease, cancer, pulmonary disease and use of blood pressure medication) in three pre-selected municipalities within the county, to perform treadmill testing with direct measurement of maximal oxygen uptake (VO_2max_). Out of 12609 potential eligible participants, 5633 appeared, and 1003 failed to complete the cardiopulmonary exercise test (CPET), withdrew or were excluded for medical reasons detected during medical interview. 4637 participants completed the exercise testing.

### Ethics statement

The study was approved by REK- Regional Committees for Medical and Health Research Ethics (2013/1788/REK nord), the Norwegian Data Inspectorate and the National Directorate of Health. The study was conducted in conformity with the Declaration of Helsinki and all participants signed a document of informed consent.

### Exercise test procedures

A 10-minute warm-up was implemented with workload individualized to induce some sweat, moderately augmented heart rate and breathing, but devoid of exhaustion. Subsequent the warm-up subjects entered the treadmill used for testing (DK7830; DK City, Taichung, Taiwan) and were equipped with a heart rate monitor (Polar S610 or RS400; Polar, Kempele, Finland) and face mask (Hans Rudolph; Shawnee, KS). Subjects were instructed to avoid handrail grasp. Cardiorespiratory variables were measured continuously using ergospirometry (MetaMax II; Cortex Biophysik GmbH, Leipzip, Germany) connected to computer software (Cortex MetaSoft, version 1.11.5). A graded individualized treadmill protocol, starting with the warm-up workload, was used with subjects walking or running at gradually increased speed and/or inclination. Treadmill speed was increased (0.5–1.0 km⋅h^-1^) when VO_2_ uptake measurements remained stable > 30 s, keeping a fixed inclination if possible. Test was terminated when subject reached volitional exhaustion (e.g. leg fatigue and shortness of breath), preferably within 8–12 minutes (Table 1). VO_2max_ was taken as the mean of the three successive highest 10-s VO_2_ values and defined by a leveling off of VO_2_ (<2 mL·kg^-1^·min^-1^ change over the span of these successive measurements) despite increasing speed and/or inclination, in combination with a respiratory exchange ratio (R) above 1.05 and subjective volitional exhaustion (e.g. leg fatigue and shortness of breath). Since a total of 17.6% of the subjects failed to reach all the criteria, the term VO_2peak_ was used. During the incremental test most subjects had their steady-state VO_2_ measured at one (n = 2827) or two (n = 2576) submaximal levels. At the first submaximal level (VO_2_ < ventilatory anaerobic threshold (established by V-slope)) steady-state VO_2_ was attained from each subject after 3 minutes. Measurements at this level were used to develop the submaximal models. At each level, as well as at peak performance, treadmill- velocity and inclination in addition to heart rate were also registered. Velocities in the range 5.9–8.0 km⋅h^-1^ typically represents the transition from walking to running, with individual variation attributed differences in e.g. stride length, leg length and body-size [20–22]. Test velocities used in development of the VO2_peak_ prediction models suggest that most participants (92%) walked during the first submaximal measurement, whereas approximately 80% were running during peak measurements. An average of 87% of all participants used 10% treadmill inclination during both the first submaximal and the peak measurements. For development of the submaximal performance models peak heart rates (HR_peak_) were predicted from age in two gender specific linear regression models based on the HUNT 3 fitness data (men: HR_peak_ = 215.336–0.73 x age, R^2^ = 0.40, SEE = 12.25 and women: HR_peak_ = 2 12.497–0.702 x age, R^2^ = 0.40, SEE = 11.71) and integrated into the fraction peak heart rate variable (Fraction peakHR: HR_submax_/215.336–0.73 x age (men); HR_submax_/212.497–0.702 x age (women)). All tests were performed by trained personnel and test equipment were routinely calibrated with volume ventilation calibrated every third test and gas calibrated every fifth test. Height was measured in centimeters with one decimal, and weight in kilograms with one decimal by internally standardized procedures.

**Table 1:** Test protocol for using the VO_2peak_ prediction models derived from treadmill work.

	Submaximal performance model*	Peak performance model**
**1.**	Warm-up: 10-minutes with individualized workload that induces some sweat, moderately increased heart rate and breathing, but devoid of exhaustion. Do not grab handrails if not necessary***.	
**2.**	Continue for 3 minutes on the established warm-up workload	Use warm-up settings (velocity and inclination) as initial workload for the 3 first minutes. Then increase velocity (0.5–1.0 km⋅h^-1^) or inclination*** approximately every minute.(Anticipated test duration: 8–12 minutes)
**3.**	Record HR_submax_, velocity and inclination after 3 minutes and use for prediction	Record velocity and inclination subsequent last workload endured > 30 seconds and use for prediction
**4.**		Walk for 5 minutes at a low workload to reduce heart rate and allow removal of accumulated lactate.

*Approximately 92% walked during the submaximal measurements

**Approximately 80% ran during peak measurements; anticipated test duration 8–12 minutes

***87% used 10% inclination as fixed slope angle during both submaximal and peak tests

### Statistical analysis

Descriptive statistics are given as mean and standard deviation for men and women, respectively. Potential variables were chosen on the basis of correlation with measured VO_2peak_ in previous literature, and entered subsequently in a hierarchical linear regression model. All the retained variables (Treadmill inclination and velocity, weight, age and Fraction HR_peak_) made a considerable influence on total model fit. The models were checked for normality and homoscedasticity of residuals and these assumptions were satisfied. All models presented in this paper were derived from the total sample. Internal cross-validation was checked by data-splitting procedures, i.e. SPSS randomly selected approximately 50% of all cases, here denoted validation sample, with the remaining cases denoted cross-validation sample. In these subsets linear regression analysis were performed on the validation sample and applied to predict VO_2peak_ in both the validation- and the cross-validation samples. Model fit was evaluated by squared multiple regression coefficients (R^2^) and standard errors of the estimate (SEE). R^2^ and R^2^ adjusted increased similarly for each new independent variable added to the models. R^2^ and R^2^ adjusted were either identical or differed in the third decimal place, showing that both had almost identical impact on the outcome variable. As a result R^2^ was chosen throughout this paper. To be able to compare the model precision to models derived from external samples we also calculated the % SEE which refers to the percentage of the measured mean VO_2peak_ within which the estimates generally fall. In the total sample, as well as subgroups of age, VO_2peak_ and treadmill velocity, we calculated constant error (CE) and total error (TE) for the model. CE represents the mean difference between measured and predicted values (∑ (measured-predicted)/n), while TE represents the squared mean differences (√∑ (measured-predicted) ^2^/n). Pearson correlation and variance explained between measured and predicted VO_2peak_ were used to examine potential shrinkage between validation and cross-validation samples. Further internal validation was done by cross-classifying subjects into quintiles of measured and predicted VO_2peak_. Measures of rank correlation and agreement were tested by use of Kendall`Tau and Cohens`Kappa statistics. Two-sided Paired Samples T-test was used to establish differences between measured and predicted VO_2peak_. Statistical analyses were performed with SPSS 20.0 (Statistical package for social sciences, Chicago, IL, USA).

## Results

Descriptive characteristics are presented in Table 2. Descriptive data in the validation and cross-validation samples were equally distributed (Table 3). Additional descriptive data of the HUNT 3 fitness population are displayed in a previous study [23].

**Table 2:** Descriptive data: The HUNT 3 fitness study.

	All	Men	Women
	(n = 4637)	(n = 2266)	(n = 2371)
Age (yr.)	49 ± 14	50 ± 14	49 ± 14
**Physical data**			
Height (cm)	172.0 ± 9.1	179.2 ± 6.5	165.7 ± 6.0
Weight (kg)	77.4 ± 13.9	85.6 ± 11.6	70.1 ± 11.6
**Physiological data**			
VO_2peak_ (L⋅min^-1^)	3.10 ± 0.91	3.75 ± 0.76	2.47 ± 0.50
VO_2peak_ (mL⋅kg^-1^⋅min^-1^)	40.0 ± 9.5	44.3 ± 9.3	35.9 ± 7.8
R (VCO_2_⋅VO_2_^-1^)	1.12 ± 0.07	1.13 ± 0.07	1.12 ± 0.07
HR_peak_ (beats⋅min^-1^)	179 ± 15	180 ± 16	179 ± 15
RHR (beats⋅min^-1^)	59 ± 10	58 ± 9	61 ± 10

Data are presented as arithmetic mean ± SD. VO_2peak_: peak oxygen uptake; HR_peak_: peak heart rate; RHR: resting heart rate.

**Table 3:** Descriptive data for the male and female validation and cross-validation sample: The HUNT 3 fitness study.

	Validation sample		Cross-validation sample	
	Men	Women	Men	Women
	(n = 1105)	(n = 1199)	(n = 1161)	(n = 1172)
Age (yr.)	50 ± 14	49 ± 14	50 ± 14	49 ± 14
**Physical data**				
Height (cm)	179.1 ± 6.5	165.6 ± 6.1	179.2 ± 6.5	165.8 ± 5.9
Weight (kg)	85.4 ± 11.7	69.8 ± 11.1	85.7 ± 11.5	70.5 ± 12.0
**Physiological data**				
VO_2peak_ (L⋅min^-1^)	3.75 ± 0.77	2.47 ± 0.51	3.75 ± 0.75	2.48 ± 0.50
VO_2peak_ (mL⋅kg^-1^⋅min^-1^)	44.4 ± 9.3	36.0 ± 7.9	44.3 ± 9.2	35.9 ± 7.6
R (VCO_2_⋅VO_2_^-1^)	1.13 ± 0.07	1.12 ± 0.08	1.13 ± 0.07	1.12 ± 0.07
HR_peak_ (beats⋅min^-1^)	180 ± 16	178 ± 15	180 ± 16	180 ± 15
RHR (beats⋅min^-1^)	58 ± 9	61 ± 10	58 ± 10	61 ± 10

Data are presented as arithmetic mean ± SD. VO_2peak_: peak oxygen uptake; HR_peak_: peak heart rate; RHR: resting heart rate.

### Predicting VO_2peak_ from peak treadmill performance

**Fig 1:**
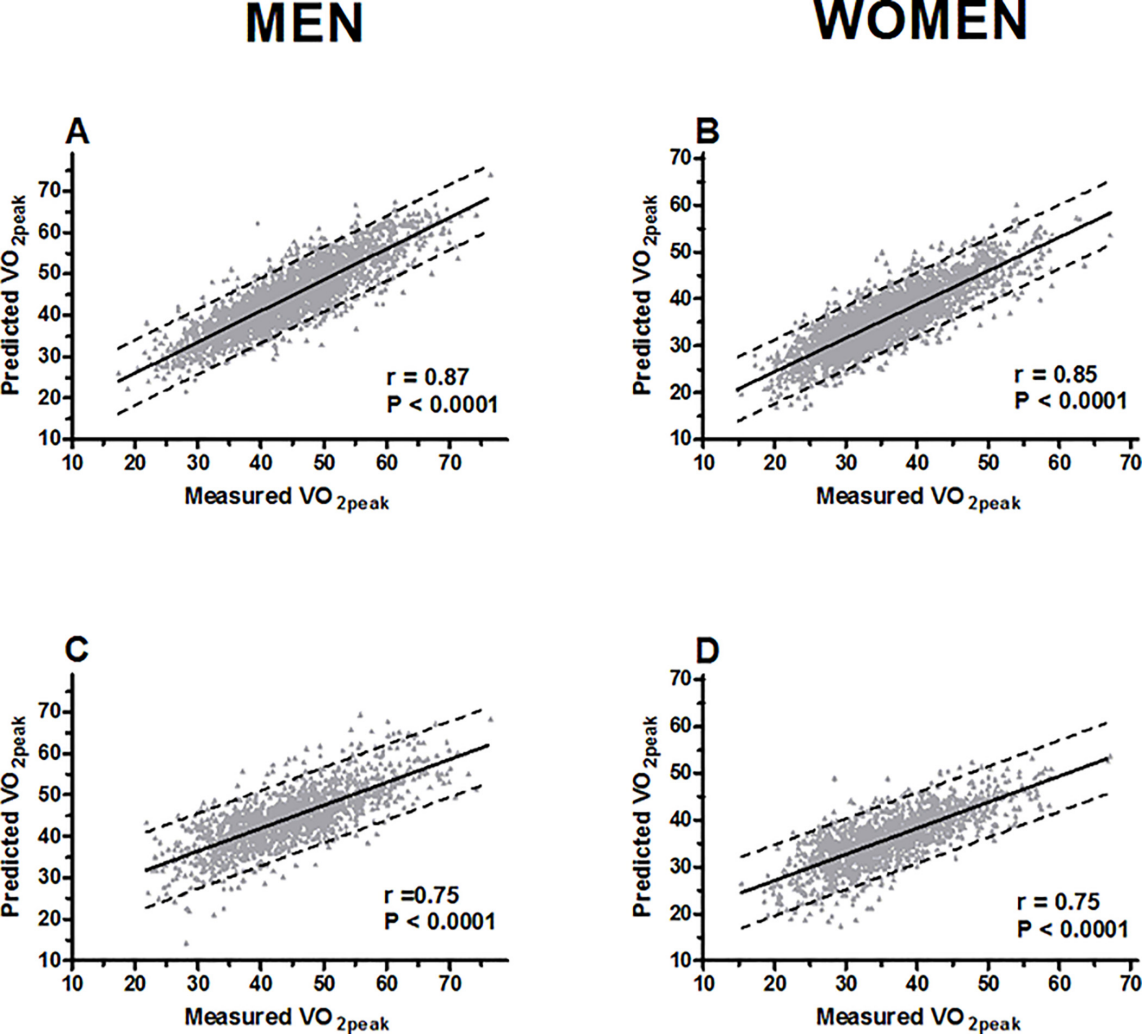
Correlation plots between measured and predicted VO_2peak_ with 95% prediction bands from peak treadmill performance (A and B), and submaximal treadmill performance (C and D).

Peak treadmill inclination and velocity accounted for most of the variance explained by the VO_2peak_ prediction model (men: R^2^ = 0.72, p<0.001; women: R^2^ = 0.68, p<0.001), with velocity being the paramount factor. Modest influence were seen from weight and age, and the total explained variance for the peak performance prediction model was R^2^ = 0.75 (p<0.001) in men and R^2^ = 0.72 (p<0.001) in women. Including resting heart rate and peak heart rate into the model did not contribute considerable changes in R^2^ and SEE and were thus excluded from the models. A strong correlation was demonstrated between the predicted- and measured VO_2peak_ (men: r = 0.87; women: r = 0.85) (Fig 1). Two gender specific VO_2peak_ prediction equations were derived from multiple linear regression using the total sample, male: VO_2peak_ = 24.24 + (0.599 x treadmill inclination in %) + (3.197 x treadmill velocity in km⋅h^-1^)–(0.122 x body weight in kilos)–(0.126 x age in years); women: VO_2peak_ = 17.21 + (0.582 x treadmill inclination in percent) + (3.317 x treadmill velocity in km⋅h^-1^)–(0.116 x weight in kilos)–(0.099 x age in years) (Tables 4, 5 and 6).

**Table 4:** Multiple linear regression coefficients for predicting VO_2peak_ (mL⋅kg^-1^⋅min^-1^) from peak measurements in total sample.

	Men			Women		
	β	Standardized β	Pearson Correlation	β	Standardized β	Pearson Correlation
Intercept	24.24			17.21		
Inclination	0.599	0.084	-0.260	0.582	0.130	-0.286
Velocity	3.197	0.729	0.842	3.317	0.717	0.818
Weight	-0.122	-0.152	-0.346	-0.116	-0.168	-0.410
Age	-0.126	-0.185	-0.572	-0.099	-0.175	-0.574
R	0.866			0.848		
R^2^	0.750			0.719		
SEE	4.63			4.11		

R: multiple regression coefficient; R^2^: coefficient of determination; SEE: standard error of estimate, Inclination (%), Velocity (km⋅h^-1^), Weight (kg), Age (yr.).

**Table 5:** Multiple linear regression analysis for predicting VO_2peak_ (mL⋅kg^-1^⋅min^-1^) from peak measurements in total sample: The HUNT 3 fitness study.

	Men						Women					
	R	R^2^	ΔR^2^	p	SEE	%SEE	R	R^2^	ΔR^2^	p	SEE	%SEE
Inclination	0.26	0.07	^__^	^__^	8.94	20.2	0.29	0.08	^__^	^__^	7.43	20.7
Inclination, Velocity	0.85	0.72	0.65	<0.001	4.94	11.2	0.83	0.68	0.60	<0.001	4.38	12.2
Inclination, Velocity, Weight	0.85	0.73	0.01	<0.001	4.83	10.9	0.84	0.70	0.02	<0.001	4.25	11.8
Inclination, Velocity, Weight, Age	0.87	0.75	0.02	<0.001	4.63	10.5	0.85	0.72	0.02	<0.001	4.11	11.5

R: multiple regression coefficient; R^2^: coefficient of determination; SEE: standard error of estimate; p: level of significance, Inclination (%), Velocity (km⋅h^-1^), Weight (kg), Age (yr.).

**Table 6:** VO_2peak_ prediction models based on peak (P) and submaximal (S) treadmill performance in men and women.

Equations	Velocity (Range)	Inclination (Range)	R^2^	SEE(%)
**Men**				
VO_2peak_ (P) = 24.24 + (0.599 x inclination) + (3.197 x velocity)–(0.122 x weight)–(0.126 x age)	9.6 ± 2.1 (3.5–17.0)	10.3 ± 1.3 (0–15)	0.75	10.5
VO_2peak_ (S) = 35.25 + (1.276 x inclination) + (6.402 x velocity)–(0.196 x weight)–(27.615 x test heart rate/215.336–0.73 x age)	5.6 ± 1.0 (2.5–12.0)	9.7 ± 1.3 (0–10)	0.55	14.1
**Women**				
VO_2peak_ (P) = 17.21 + (0.582 x inclination) + (3.317 x velocity)–(0.116 x weight)–(0.099 x age)	7.6 ± 1.7 (2.5–14.0)	10.7 ± 1.7 (0–16)	0.72	11.5
VO_2peak_ (S) = 23.77 + (1.205 x inclination) + (6.051 x velocity)–(0.16 x weight)–(20.671 x test heart rate/212.497–0.702 x age)	5.0 ± 0.8 (2.0–8.0)	9.3 ± 1.8 (0–15)	0.56	14.4

VO_2peak_: peak oxygen uptake, velocity (km⋅h^-1^) and inclination (%) are presented as arithmetic mean ± SD, Range (minimum-maximum), age (yr.), weight (kg), R^2^: coefficient of determination, SEE: standard error of estimate.

### Cross-validation of the peak performance prediction model

The Coefficient of determination (R^2^) remained stable between the total sample (0.75 and 0.72) and the validation sample (0.76 and 0.72) among both men and women, respectively (Tables 5 and 7), thus suggesting an internally robust prediction model. Also, there were non-significant differences between measured and predicted VO_2peak_, and we display CE values close to zero, in the total sample (− 0 .03 and 0.02), validation sample (– 0.03 and– 0.03) and cross-validation sample (− 0.21 and − 0.04) in both men and women, respectively (Tables 8–10), signifying a valid prediction of the mean VO_2peak_ without systematical over- or under prediction, respectively. Our prediction models continue to be stable when stratified into subgroups of age and treadmill velocity (non-significant differences between measured and predicted VO_2peak_). However, when divided into VO_2peak_ subgroups the least fit participants (<35 mL⋅kg^-1^⋅min^-1^ for men and <30 mL⋅kg^-1^⋅min^-1^ for women) tended to be overestimated (men: p<0.001; women: p<0.001), and the most fit participants (>50 mL⋅kg^-1^⋅min^-1^ for men and >40 mL⋅kg^-1^⋅min^-1^ for women) tended to be underestimated (men: p<0.001; women: p<0.001). This is in agreement with the validation and cross-validation samples. The least fit men and women showed CE values of– 3.50 and– 2.60 vs. most fit 2.73 and 2.81, respectively, with corresponding TE values of 5.01 and 4.35 vs. 5.27 and 4.80, with similar tendencies in validation and cross-validation samples. In the medium fit participants (VO_2peak_ between 35 and 50 mL⋅kg^-1^⋅min^-1^ for men and between 30 and 40 mL⋅kg^-1^⋅min^-1^ for women) the model appears to predict VO_2peak_ fairly well (men: p<0.05; women: p<0.001), with CE values in men and women of– 0.28 and– 0.37, respectively. The same tendencies are shown in the validation and cross-validation samples (Tables 8–10). Pearson correlation showed minimal shrinkage between validation- sample (men: r = 0.870, R^2^ = 0.757, p < 0.01; women: r = 0.846, R^2^ = 0.716, p < 0.01) and cross-validation sample (men: r = 0.863, R^2^ = 0.745, p = 0.01; women: r = 0.850, R^2^ = 0.723, p < 0.01). Thus, the entire sample was used in development of the models.

**Table 7:** Multiple linear regression analysis for predicting VO_2peak_ (mL⋅kg^-1^⋅min^-1^) from peak measurements in men and women, validation samples: The HUNT 3 fitness study.

	Validation sample men						Validation sample women					
	R	R^2^	ΔR^2^	p	SEE	%SEE	R	R^2^	ΔR^2^	p	SEE	%SEE
Inclination	0.23	0.05	^__^	^__^	9.09	20.5	0.26	0.07	^__^	^__^	7.62	21.2
Inclination, Velocity	0.85	0.73	0.68	<0.001	4.88	11.0	0.83	0.68	0.62	<0.001	4.45	12.4
Inclination, Velocity, Weight	0.86	0.74	0.01	<0.001	4.78	10.8	0.84	0.70	0.02	<0.001	4.33	12.0
Inclination, Velocity, Weight, Age	0.87	0.76	0.02	<0.001	4.61	10.4	0.85	0.72	0.02	<0.001	4.21	11.7

R: multiple regression coefficient; R^2^: coefficient of determination; SEE: standard error of estimate; p: level of significance, Inclination (%), Velocity (km⋅h^-1^), Weight (kg), Age (yr.).

**Table 8:** Measured vs. Predicted VO_2peak_, from peak measurements, in the total sample: The HUNT 3 fitness study.

	Men						Women					
	Measured VO_2peak_	Predicted VO_2peak_	CE	%SEE	%TE	N (%)	Measured VO_2peak_	Predicted VO_2peak_	CE	%SEE	%TE	N (%)
**Age (yr.)**												
<40	50.9 ± 8.5	51.1 ± 7.2	-0.25	9.0	9.0	550 (24.4)	41.0 ± 7.4	41.1 ± 6.0	-0.09	10.5	10.5	664 (28.0)
40-60	44.1 ± 8.1	44.0 ± 6.6	0.10	10.4	10.4	1231 (54.4)	35.6 ± 6.8	35.6 ± 5.2	0.06	11.4	11.4	1249 (52.8)
>60	37.3 ± 7.4	37.4 ± 5.7	-0.09	12.6	12.6	479 (21.2)	29.4 ± 5.4	29.4 ± 4.3	0.06	13.0	13.4	455 (19.2)
**VO**_**2peak**_												
<35(<30)	31.2 ± 3.2	34.7 ± 4.1	-3.50	8.6	16.1	388 (17.2)	26.4 ± 2.8	29.0 ± 4.0	-2.60	8.9	16.5	547 (23.1)
35-50 (30-40)	42.8 ± 4.2	43.0 ± 5.2	-0.28	7.7	9.7	1266 (56.0)	34.9 ± 2.9	35.2 ± 4.0	-0.37	7.0	10.1	1154 (48.7)
>50(>40)	56.0 ± 5.0	53.3 ± 5.6	2.73	6.8	9.4	607 (26.8)	45.6 ± 4.8	42.8 ± 5.0	2.81	7.7	10.5	668 (28.2)
**Velocity**												
<7	31.9 ± 5.3	32.1 ± 3.2	-0.17	13.1	13.1	209 (9.3)	29.3 ± 4.7	29.2 ± 3.4	0.15	12.4	12.5	757 (31.9)
7-10	41.0 ± 6.2	41.0 ± 4.0	0.05	11.2	11.2	1260 (55.7)	37.9 ± 5.9	38.0 ± 3.9	-0.10	11.3	11.3	1459 (61.6)
>10	52.9 ± 6.7	53.0 ± 4.8	-0.11	9.1	9.1	792 (35.0)	50.1 ± 5.4	49.6 ± 3.2	0.47	8.9	8.9	153 (6.5)
**Total**	44.3 ± 9.3	44.3 ± 8.0	-0.03	10.5	10.5	2261	35.9 ± 7.8	35.9 ± 6.6	0.02	11.5	11.5	2369

VO_2peak_ were categorized into 3 groups from measured VO_2peak_; <35, 35–50, >50 (mL kg^-1^ min^-1^) and <30, 30–40, >40 (mL kg^-1^ min^-1^) were cutoff values for men and women, respectively; velocity: treadmill velocity (km h^-1^).

**Table 9:** Cross-validation of Measured vs. Predicted VO_2peak_, from peak measurements, in men: The HUNT 3 fitness study.

	Validation sample						Cross-validation sample					
	Measured VO_2peak_	Predicted VO_2peak_	CE	%SEE	%TE	N (%)	Measured VO_2peak_	Predicted VO_2peak_	CE	%SEE	%TE	N (%)
**Age (yr.)**												
<40	50.9 ± 8.6	51.2 ± 7.2	-0.34	8.6	8.6	274 (24.8)	50.8 ± 8.4	51.2 ± 7.8	-0.43	9.3	9.5	276 (23.8)
40-60	43.9 ± 8.0	43.8 ± 6.6	0.14	10.4	10.4	610 (55.4)	44.3 ± 8.3	44.5 ± 6.9	-0.17	10.5	10.5	621 (53.6)
>60	37.4 ± 8.1	37.5 ± 6.2	-0.07	13.2	13.2	218 (19.8)	37.3 ± 6.8	37.4 ± 5.7	-0.10	12.1	12.2	261 (22.6)
**VO**_**2peak**_												
<35	31.0 ± 3.4	34.5 ± 4.3	-3.54	8.5	16.0	189 (17.2)	31.4 ± 3.0	34.6 ± 4.1	-3.20	8.5	15.7	199 (17.2)
35-50	42.9 ± 4.3	43.1 ± 5.2	-0.26	7.8	9.8	616 (55.8)	42.7 ± 4.1	43.1 ± 5.4	-0.40	7.6	10.1	650 (56.1)
>50	56.0 ± 5.0	53.3 ± 5.7	2.70	6.5	9.2	297 (27.0)	56.0 ± 5.0	53.9 ± 5.7	2.10	7.1	9.3	310 (26.7)
**Velocity**												
<7	31.0 ± 5.8	31.4 ± 3.5	-0.44	15.0	15.0	93 (8.4)	32.7 ± 4.8	32.2 ± 3.0	0.54	11.3	11.3	116 (10.0)
7-10	40.9 ± 6.2	40.9 ± 4.0	0.11	11.4	11.4	621 (56.4)	41.0 ± 6.2	41.0 ± 4.1	0.04	11.0	11.0	639 (55.1)
>10	53.0 ± 6.5	53.1 ± 4.7	- 0.14	8.4	8.4	388 (35.2)	52.8 ± 7.0	53.6 ± 5.0	-0.82	9.7	9.8	404 (34.9)
**Total**	44.4 ± 9.3	44.4 ± 8.1	-0.03	10.4	10.4	1102	44.3 ± 9.2	44.5 ± 8.3	-0.21	10.5	10.5	1159

VO_2peak_ were categorized into 3 groups from measured VO_2peak_; <35, 35–50, >50 (mL kg^-1^ min^-1^) were cutoff values for men; velocity: treadmill velocity (km h^-1^).

**Table 10:** Cross-validation of Measured vs. Predicted VO_2peak_, from peak measurements, in women: The HUNT 3 fitness study.

	Validation sample						Cross-validation sample					
	Measured VO_2peak_	Predicted VO_2peak_	CE	%SEE	%TE	N (%)	Measured VO_2peak_	Predicted VO_2peak_	CE	%SEE	%TE	N (%)
**Age (yr.)**												
<40	41.2 ± 7.4	41.5 ± 5.9	-0.27	10.9	10.9	337 (28.1)	40.7 ± 7.4	40.7 ± 6.1	0.03	10.1	10.1	327 (28.0)
40-60	35.8 ± 6.8	35.6 ± 5.0	0.18	11.5	11.6	628 (52.4)	35.5 ± 6.7	35.6 ± 5.4	-0.16	11.2	12.2	621 (53.1)
>60	29.0 ± 5.4	29.2 ± 4.6	-0.23	13.3	13.6	233 (19.5)	29.9 ± 5.4	29.7 ± 4.2	0.23	13.2	13.3	222 (18.9)
**VO**_**2peak**_												
<30	26.2 ± 2.7	28.8 ± 4.1	-2.60	8.8	16.8	279 (23.3)	26.6 ± 2.8	29.2 ± 4.0	-2.60	8.9	16.3	268 (22.9)
30-40	34.9 ± 2.9	35.3 ± 4.0	-0.44	7.1	10.1	575 (48.0)	34.8 ± 2.9	35.2 ± 4.1	-0.37	7.0	10.2	579 (49.5)
>40)	45.7 ± 4.8	43.0 ± 4.9	2.73	8.2	10.9	344 (28.7)	45.5 ± 4.7	42.8 ± 5.3	2.69	6.9	9.9	324 (27.6)
**Velocity**												
<7	29.2 ± 4.8	29.1 ± 3.6	0.07	12.6	12.8	392 (32.7)	29.4 ± 4.6	29.2 ± 3.4	0.22	12.1	12.3	365 (31.2)
7-10	38.2 ± 6.1	38.2 ± 3.9	-0.02	11.4	11.4	725 (60.5)	37.6 ± 5.7	37.8 ± 4.0	-0.26	11.2	11.2	734 (62.6)
>10	49.1 ± 5.7	49.5 ± 3.0	-0.38	9.7	9.7	81 (6.8)	51.3 ± 4.8	50.3 ± 3.5	0.99	7.5	7.8	72 (6.2)
**Total**	36.0 ± 7.9	36.0 ± 6.7	-0.03	11.7	11.7	1198	35.9 ± 7.6	35.9 ± 6.6	-0.04	11.2	11.2	1171

VO_2peak_ were categorized into 3 groups from measured VO_2peak_; <30, 30–40, >40 (mL kg^-1^ min^-1^) were cutoff values for women; velocity: treadmill velocity (km h^-1^).

### Cross-classification of participants in the peak performance prediction model

The models managed to categorize participants fairly accurately into the correct measured VO_2peak_ group when cross-classifying participants into quintiles of measured and predicted VO_2peak_ (Table 11). In total, 75.3% and 77.6% of the men and women, predicted to be in the lowest quintile, were classified correctly into the lowest measured quintile, respectively, while 95.4% and 96.7% were correctly classified within the correct or closest measured quintile. 77.4% and 78.0% of the men and women, predicted to be in the highest quintile, were correctly classified into the highest measured quintile, respectively, with 95.8% and 95.9% being classified correctly into one of the two highest quintiles (Table 11). The rank correlation between measured and predicted quintiles were 0.74 and 0.70 in men and women, respectively, while measure of agreement by Kappa statistic was 0.45 in men and 0.41 in women.

**Table 11:** Cross-tabulation between measured and predicted VO_2peak_ quintiles from peak performance for men and women.

	Men						Women					
	Measured VO_2peak_						Measured VO_2peak_					
Predicted	Q_1_	Q_2_	Q_3_	Q_4_	Q_5_	Total	Q_1_	Q_2_	Q_3_	Q_4_	Q_5_	Total
VO_2peak_												
**Q**_**1**_	259	69	14	2	0	344	256	63	10	1	0	330
	75.3%	20.1%	4.1%	0.6%	0%	100%	77.6%	19.1%	3.0%	0.3%	0%	100%
**Q**_**2**_	169	232	108	30	3	542	158	216	134	30	5	543
	31.2%	42.8%	19.9%	5.5%	0.6%	100%	29.1%	39.8%	24.7%	5.5%	0.9%	100%
**Q**_**3**_	19	132	206	123	22	502	53	152	208	127	29	569
	3.8%	26.3%	41.0%	24.5%	4.4%	100%	9.3%	26.7%	36.6%	22.3%	5.1%	100%
**Q**_**4**_	4	18	110	221	108	461	6	40	109	246	135	536
	0.9%	3.9%	23.9%	47.9%	23.4%	100%	1.1%	7.5%	20.3%	45.9%	25.2%	100%
**Q**_**5**_	0	2	15	76	319	412	1	2	13	70	305	391
	0%	0.5%	3.6%	18.4%	77.4%	100%	0.3%	0.5%	3.3%	17.9%	78.0%	100%
**Total**	451	453	453	452	452	2261	474	473	474	474	474	2369

Q_1-5_, quintile cut-off values for measured and predicted VO_2peak_. Men: <36.0, 36.0–41.5, 41.6–46.5, 46.6–52.0, >52.0 (measured quintiles); and <37.1, 37.1–41.6, 41.7–46.0, 46.1–51.3, >51.3 (predicted quintiles). Women: <29.1, 29.1–33.3, 33.4–37.3, 37.4–42.1, <42.1 (measured quintiles), and <30.5, 30.5–33.8, 33.9–37.1, 37.2–41.2, >41.2 (predicted quintiles). Kendall`s Tau statistic: 0.74 for men and 0.70 for women. Cohen Kappa statistic: 0.45 in men and 0.41 in women.

### Predicting VO_2peak_ from submaximal treadmill performance

Treadmill inclination and velocity accounted for the major part of the explained variance in predicting VO_2peak_ (men: R^2^ = 0.40, p<0.001; women: R^2^ = 0.43, p<0.001), with velocity being the most important variable (men: R^2^ = 0.35, p<0.001; women: R^2^ = 0.34, p<0.001). Weight (men: R^2^ = 0.06, p<0.001; women: R^2^ = 0.06, p<0.001) and fraction peakHR (HR_submax_ and age, men: R^2^ = 0.09, p<0.001; women: R^2^ = 0.06, p<0.001) accounted for a lesser but considerable part of the total variance (men: R^2^ = 0.55, p<0.001; women: R^2^ = 0.56, p<0.001). Resting heart rate yielded negligible changes in R^2^ and SEE and hence excluded from the prediction model. A high correlation (r = 0.75) was observed between predicted- and measured VO_2peak_ among men and women (Fig 1). The final regression models derived from total sample were 35.25 + (1.276 x treadmill inclination in %) + (6.402 x velocity in km⋅h^-1^)–(0.196 x weight in kilos)–(27.615 x HR_submax_/215.336–0.73 x age in years) for men and 23.77 + (1.205 x treadmill inclination in %) + (6.051 x velocity in km⋅h^-1^)–(0.160 x weight in kilos)–(20.671 x HR_submax_/212.497–0.702 x age in years) for women (Tables 6, 12 and 13).

**Table 12:** Multiple linear regression coefficients for predicting VO_2peak_ (mL⋅kg^-1^⋅min^-1^) from submaximal measurements in total sample.

	Men			Women		
	β	Standardized β	Pearson Correlation	β	Standardized β	Pearson Correlation
Intercept	35.25			23.77		
Inclination	1.276	0.200	0.233	1.205	0.307	0.300
Velocity	6.402	0.662	0.612	6.051	0.606	0.612
Weight	-0.196	-0.240	-0.346	-0.160	-0.228	-0.410
Fraction HR_peak_	-27.615	-0.280	-0.014	-20.671	-0.253	-0.040
R	0.744			0.746		
R^2^	0.554			0.557		
SEE	6.24			5.17		

Fraction HR_peak_: fraction of peak heart rate; R: multiple regression coefficient; R^2^: coefficient of determination; SEE: standard error of estimate, Inclination (%), Velocity (km⋅h^-1^), Weight (kg).

**Table 13:** Multiple linear regression analysis for predicting VO_2peak_ from submaximal measurements on total sample: The HUNT 3 fitness study.

	Men						Women					
	R	R^2^	ΔR^2^	p	SEE	%SEE	R	R^2^	ΔR^2^	p	SEE	%SEE
Inclination	0.23	0.05	^__^	^__^	9.00	20.3	0.30	0.09	^__^	^__^	7.34	20.4
Inclination, Velocity	0.63	0.40	0.35	<0.001	7.16	16.2	0.66	0.43	0.34	<0.001	5.81	16.2
Inclination, Velocity, Weight	0.68	0.46	0.06	<0.001	6.77	15.3	0.70	0.49	0.06	<0.001	5.48	15.2
Inclination, Velocity, Weight, Fraction HR_peak_	0.74	0.55	0.09	<0.001	6.24	14.1	0.75	0.56	0.06	<0.001	5.17	14.4

Fraction HR_peak_: fraction of peak heart rate; R: multiple regression coefficient; R^2^: coefficient of determination; SEE: standard error of estimate; p: level of significance, Inclination (%), Velocity (km⋅h^-1^), Weight (kg).

### Cross-validation of the submaximal performance prediction model

R^2^ was stable between the total sample (0.55 and 0.56) and validation sample (0.54 and 0.56) in men and women, respectively (Tables 13 and 14), indicating a strong prediction model. Furthermore, there were non-significant differences between the measured and predicted VO_2peak_, and CE was close to zero in the total sample (− 0.14 and − 0.12), validation sample (− 0.27 and − 0.03) and cross-validation sample (− 0.09 and − 0.11), among both men and women, respectively (Tables 15–17), thus suggesting a valid estimation of mean VO_2peak_. The submaximal prediction models are less stable when stratified into subgroups of age, VO_2peak_ and treadmill velocity groups. There was a tendency towards underestimating VO_2peak_ (p<0.001 and p<0.001, with CE 3.03 and 1.46 among men and women, respectively, TE 6.78 and 5.63) in the youngest subjects (<40 years), with a subsequent overestimation (p<0.001 and p<0.001, with CE– 2.88 and– 1.70 among men and women, respectively, TE 6.42 and 4.86) in the oldest subjects (>60 years), whereas VO_2peak_ was predicted fairly well (non-significant difference and p<0.01, with CE– 0.41 and– 0.58 among men and women, respectively, TE 5.90 and 5.02) in middle age group (40–60 years). In the VO_2peak_ subgroups there was an apparent overestimation (p<0.001 and p<0.001, with CE– 5.82 and– 4.37 among men and women, respectively, TE 8.04 and 6.12) in the least fit groups (<35 mL⋅kg^-1^⋅min^-1^ for men and <30 mL⋅kg^-1^⋅min^-1^ for women), with a succeeding underestimation (p<0.001 and p<0.001, with CE 5.30 and 4.10 among men and women, respectively, TE 7.41 and 5.94) in the most fit group (>50 mL⋅kg^-1^⋅min^-1^ for men and >40 mL⋅kg^-1^⋅min^-1^ for women). However, the submaximal models predicted VO_2peak_ fairly well (p<0.001 and p<0.001, with CE– 0.79 and– 0.53 among men and women, respectively, TE 4.83 and 4.07) in the medium fit subjects (VO_2peak_ in the range of 35–50 mL⋅kg^-1^⋅min^-1^ for men and between 30 and 40 mL⋅kg^-1^⋅min^-1^ for women). All velocity groups predicted VO_2peak_ fairly well, with no significant differences between measured and predicted VO_2peak_ (<5 km⋅h^-1^: CE– 0.44 and– 0.49 among men and women, respectively, and TE 5.80 and 4.80; 5–6 km⋅h^-1^: CE– 0.35 and– 0.24, TE 6.24 and 5.28; > 6 km⋅h^-1^: CE– 0.36 and– 0.91, with TE 6.54 and 6.96). Findings in the total sample are in agreement with the overall tendencies in the validation and cross-validation samples (Tables 15–17). Pearson correlation showed minor shrinkage between validation- sample (men: r = 0.733, R^2^ = 0.537, p < 0.01; women: r = 0.749, R^2^ = 0.561, p < 0.01) and cross-validation sample (men: r = 0.755, R^2^ = 0.570, p = 0.01; women: r = 0.743, R^2^ = 0.552, p < 0.01). Hence, the entire sample was utilized in development of the models.

**Table 14:** Multiple linear regression analysis for predicting VO_2peak_ from submaximal measurements in men and women from validation samples: The HUNT 3 fitness study.

	Validation sample men						Validation sample women					
	R	R^2^	ΔR^2^	p	SEE	%SEE	R	R^2^	ΔR^2^	p	SEE	%SEE
Inclination	0.25	0.06	^__^	^__^	8.78	19.8	0.31	0.10	^__^	^__^	7.33	20.4
Inclination, Velocity	0.62	0.38	0.32	<0.001	7.13	16.1	0.66	0.44	0.34	<0.001	5.75	16.0
Inclination, Velocity, Weight	0.67	0.45	0.07	<0.001	6.75	15.2	0.71	0.50	0.06	<0.001	5.44	15.1
Inclination, Velocity, Weight, Fraction HR_peak_	0.73	0.54	0.09	<0.001	6.21	14.0	0.75	0.56	0.06	<0.001	5.21	14.5

Fraction HR_peak_: fraction of peak heart rate; R: multiple regression coefficient; R^2^: coefficient of determination; SEE: standard error of estimate; p: level of significance, Inclination (%), Velocity (km⋅h^-1^), Weight (kg).

**Table 15:** Measured vs. Predicted VO_2peak_, from submaximal measurements, in the total sample: The HUNT 3 fitness study.

	Men						Women					
	Measured VO_2peak_	Predicted VO_2peak_	CE	%SEE	%TE	N (%)	Measured VO_2peak_	Predicted VO_2peak_	CE	%SEE	%TE	N (%)
**Age (yr.)**												
<40	50.8 ± 8.5	47.8 ± 7.0	3.22	11.4	13.4	291 (21.9)	41.0 ± 7.4	39.5 ± 5.4	1.46	13.0	13.7	371 (27.1)
40-60	44.1 ± 8.1	44.6 ± 6.2	-0.41	13.4	13.4	726 (54.5)	35.6 ± 6.8	36.3 ± 4.9	-0.58	14.0	14.1	717 (52.4)
>60	37.3 ± 7.4	40.2 ± 6.4	-2.88	15.1	17.2	315 (23.6)	29.4 ± 5.4	31.1 ± 5.0	-1.68	14.3	16.5	281 (20.5)
**VO**_**2peak**_												
<35(<30)	31.2 ± 3.2	37.0 ± 5.6	-5.82	9.4	25.8	218 (16.4)	26.4 ± 2.8	30.8 ± 4.9	-4.25	9.3	23.2	308 (22.5)
35-50 (30-40)	42.8 ± 4.2	43.5 ± 5.0	-0.79	8.8	11.3	749 (56.2)	34.9 ± 2.9	35.4 ± 4.2	-0.53	7.5	11.7	667 (48.7)
>50 (>40)	56.0 ± 5.0	50.7 ± 5.7	5.34	7.7	13.2	366 (27.4)	45.6 ± 4.8	41.5 ± 4.1	4.21	8.8	13.0	394 (28.8)
**Velocity**												
<5	35.4 ± 6.5	36.0 ± 5.0	-0.44	15.4	16.4	216 (16.2)	31.0 ± 5.6	31.5 ± 4.4	-0.49	15.0	15.5	535 (39.1)
5-6	43.9 ± 7.9	44.2 ± 4.4	-0.35	14.2	14.2	911 (68.3)	38.3 ± 6.9	38.5 ± 4.1	-0.24	13.7	13.8	787 (57.5)
>6	52.9 ± 8.0	53.2 ± 5.6	-0.36	12.2	12.4	206 (15.5)	46.7 ± 7.6	47.6 ± 3.7	-0.91	14.9	14.9	47 (3.4)
**Total**	44.3 ± 9.3	44.4 ± 6.9	-0.14	14.1	14.1	1333	35.9 ± 7.8	36.1 ± 5.8	-0.12	14.4	14.4	1369

VO_2peak_ were categorized into 3 groups from measured VO_2peak_; <35, 35–50, >50 (mL kg^-1^ min^-1^) and <30, 30–40, >40 (mL kg^-1^ min^-1^) were cutoff values for men and women, respectively; velocity: treadmill velocity (km h^-1^).

**Table 16:** Cross-validation of Measured vs. Predicted VO_2peak_, from submaximal measurements in men: The HUNT 3 fitness study.

	Validation sample						Cross-validation sample					
	Measured VO_2peak_	Predicted VO_2peak_	CE	%SEE	%TE	N (%)	Measured VO_2peak_	Predicted VO_2peak_	CE	%SEE	%TE	N (%)
**Age (yr.)**												
<40	50.9 ± 8.2	47.9 ± 6.7	3.14	10.7	12.5	142 (21.6)	50.7 ± 8.8	47.5 ± 6.9	3.44	12.4	14.0	150 (22.1)
40-60	43.9 ± 8.1	44.9 ± 5.9	-1.03	14.0	14.0	362 (55.5)	44.3 ± 8.1	45.0 ± 5.9	-0.62	12.7	12.7	364 (53.5)
>60	37.8 ± 7.1	40.7 ± 6.5	-2.86	13.7	16.0	149 (22.9)	36.9 ± 7.7	40.2 ± 6.1	-2.97	16.4	18.6	166 (24.4)
**VO**_**2peak**_												
<35	31.4 ± 3.1	37.4 ± 5.4	-5.94	9.0	25.4	102 (15.6)	31.0 ± 3.2	37.2 ± 5.6	-6.32	9.9	27.2	116 (17.1)
35-50	42.8 ± 4.1	43.8 ± 4.8	-0.93	8.9	11.4	374 (57.3)	42.7 ± 4.3	43.5 ± 4.7	-0.81	8.7	10.7	375 (55.1)
>50	55.8 ± 5.1	50.5 ± 5.6	5.41	7.5	13.2	177 (27.1)	56.2 ± 4.9	50.4 ± 5.2	5.69	7.9	13.4	189 (27.8)
**Velocity**												
<5	36.0 ± 6.7	37.1 ± 4.9	-0.86	14.4	16.3	97 (14.9)	34.9 ± 6.3	36.2 ± 5.0	-1.14	15.5	16.7	119 (17.5)
5-6	43.8 ± 7.9	44.2 ± 4.5	-0.37	14.0	14.0	456 (69.8)	43.9 ± 7.9	44.4 ± 4.1	-0.42	14.4	14.5	455 (66.9)
>6	52.8 ± 8.0	54.0 ± 5.6	-1.14	12.6	12.7	100 (15.3)	52.9 ± 8.2	53.9 ± 5.5	-1.01	11.9	12.1	106 (15.6)
**Total**	44.3 ± 9.1	44.6 ± 6.7	-0.27	14.0	14.0	653	44.3 ± 9.5	44.4 ± 6.7	-0.09	14.2	14.2	680

VO_2peak_ were categorized into 3 groups from measured VO_2peak_; <35, 35–50, >50 (mL kg^-1^ min^-1^) and <30, 30–40, >40 (mL kg^-1^ min^-1^) were cutoff values for men and women, respectively; velocity: treadmill velocity (km h^-1^).

**Table 17:** Cross-validation of Measured vs. Predicted VO_2peak_, from submaximal measurements in women: The HUNT 3 fitness study.

	Validation sample						Cross-validation sample					
	Measured VO_2peak_	Predicted VO_2peak_	CE	%SEE	%TE	N (%)	Measured VO_2peak_	Predicted VO_2peak_	CE	%SEE	%TE	N (%)
**Age (yr.)**												
<40	41.1 ± 7.3	39.5 ± 5.1	1.68	13.1	14.0	187 (27.4)	40.8 ± 7.5	39.7 ± 5.5	1.16	12.8	13.3	184 (26.8)
40-60	35.5 ± 6.7	36.3 ± 4.9	-0.67	13.8	13.9	346 (50.6)	35.8 ± 6.8	36.4 ± 4.8	-0.48	14.3	14.4	371 (54.1)
>60	29.3 ± 5.7	30.7 ± 5.0	-1.37	15.1	17.0	150 (22.0)	29.7 ± 5.1	31.6 ± 5.0	-1.82	13.8	15.8	131 (19.1)
**VO**_**2peak**_												
<30)	26.3 ± 2.8	30.6 ± 4.9	-4.23	9.5	23.7	164 (24.0)	26.5 ± 2.7	31.1 ± 4.9	-4.41	8.9	22.9	144 (21.0)
30-40	34.9 ± 2.8	35.4 ± 4.2	-0.43	7.3	11.5	320 (46.9)	34.8 ± 2.9	35.5 ± 4.1	-0.77	7.6	11.9	347 (50.6)
>40	45.6 ± 4.8	41.5 ± 4.0	4.08	8.9	12.9	199 (29.1)	45.6 ± 4.7	41.6 ± 4.1	4.24	8.7	13.0	195 (28.4)
**Velocity**												
<5	30.8 ± 5.5	31.4 ± 4.4	-0.56	15.3	16.0	273 (40.0)	31.3 ± 5.7	31.7 ± 4.2	-0.38	14.6	14.8	262 (38.2)
5-6	38.3 ± 6.7	38.5 ± 4.2	-0.16	13.6	13.8	386 (56.5)	38.3 ± 7.0	38.7 ± 3.9	-0.39	13.7	13.8	401 (58.4)
>6	47.1 ± 7.9	47.0 ± 4.1	0.19	13.5	14.2	24 (3.5)	46.3 ± 7.4	48.5 ± 3.2	-2.10	15.4	15.6	23 (3.4)
**Total**	35.9 ± 7.8	36.0 ± 5.9	-0.03	14.5	14.5	683	36.1 ± 7.7	36.3 ± 5.7	-0.11	14.2	14.3	686

VO_2peak_ were categorized into 3 groups from measured VO_2peak_; <35, 35–50, >50 (mL kg^-1^ min^-1^) and <30, 30–40, >40 (mL kg^-1^ min^-1^) were cutoff values for men and women, respectively; velocity: treadmill velocity (km h^-1^).

### Cross-classification of participants in the submaximal performance prediction model

Cross-classification of predicted (from submaximal performance) and measured VO_2peak_ achieved a fairly accurate placing of subjects into the correct VO_2peak_ quintile (Table 18). In total, 62.0% and 50.3% of the men and women were predicted appropriately into the lowest measured quintile, respectively, with an increase to 91.3% and 80.9% within the closest measured quintiles. A total of 59.0% and 72.5% of the men and women, in the highest predicted quintile, were correctly categorized into the highest measured quintile, respectively, increasing to 84.9% and 89.0% within one of the two highest quintiles (Table 18). The rank correlation between measured and predicted quintiles were 0.61 and 0.60 in men and women, respectively, while measure of agreement by Kappa statistic was 0.30 in men and 0.28 in women.

**Table 18:** Cross-tabulation between measured and predicted VO_2peak_ quintiles from submaximal performance in men and women.

	Men						Women					
	Measured VO_2peak_						Measured VO_2peak_					
Predicted	Q_1_	Q_2_	Q_3_	Q_4_	Q_5_	Total	Q_1_	Q_2_	Q_3_	Q_4_	Q_5_	Total
VO_2peak_												
**Q**_**1**_	127	60	16	2	0	205	171	104	49	15	1	340
	62.0%	29.3%	7.8%	1.0%	0%	100%	50.3%	30.6%	14.4%	4.4%	0.3%	100%
**Q**_**2**_	71	74	48	23	5	221	63	102	88	48	11	312
	32.1%	33.5%	21.7%	10.4%	2.3%	100%	20.2%	32.7%	28.2%	15.4%	3.5%	100%
**Q**_**3**_	39	81	75	61	11	267	23	52	79	97	38	289
	14.6%	30.3%	28.1%	22.8%	4.1%	100%	8.0%	18.0%	27.3%	33.6%	13.1%	100%
**Q**_**4**_	18	44	84	111	51	308	6	16	38	82	86	228
	5.8%	14.3%	27.3%	36.0%	16.6%	100%	2.6%	7.0%	16.7%	36.0%	37.7%	100%
**Q**_**5**_	3	13	34	86	196	332	1	5	16	33	145	200
	0.9%	3.9%	10.2%	25.6%	59.0%	100%	0.5%	2.5%	8.0%	16.5%	72.5%	100%
**Total**	258	272	257	283	263	1333	264	279	270	275	281	1369

Q_1-5_, quintiles cut-off values of measured and predicted VO_2peak_. Men: <36.0, 36.0–41.5, 41.6–46.5, 46.6–52.0, >52.0 (measured quintiles); and <39.0, 39.0–42.9, 43.0–46.0, 46.1–49.5, >49.5 (predicted quintiles). Women: <29.1, 29.1–33.3, 33.4–37.3, 37.4–42.1, <42.1 (measured quintiles), and <31.4, 31.4–34.8, 34.9–37.5, 37.6–40.7, >40.7 (predicted quintiles). Kendall Tau statistic: 0.61 for men and 0.60 for women. Cohen Kappa statistics: 0.30 for men and 0.28 for women.

## Discussion

The exercise-based prediction models generated in this study accurately placed approximately 91% of the low- and high-fit participants within the correct or nearest quintile of measured VO_2peak_, and predicted VO_2peak_ with fair precision using both the peak performance and submaximal models.

### Accuracy of the VO_2peak_ prediction models

The peak performance models displayed accuracy (SEE) of 10.5% (R^2^ = 0.75) and 11.5% (R^2^ = 0.72), in men and women, respectively. This is better than some previous research reporting accuracy in the range 13.3–16.6% [14,24], and also less accurate or equal to that reported by yet others (4.5–11.4%) [10–13,15,18,25]. Better prediction accuracy in other models may partly be attributed their homogeneous fitness level in sample subjects [10,12,13,15,18] and/or narrow age range [10–12]. Validating other models using HUNT 3 data is difficult given the use of different independent variables, e.g. watts on cycle ergometer [13,18,25] or 20m-shutle run [10–12]. Although the ACSM running model [20] used, similar to us, speed and gradient, it is developed from steady-state submaximal aerobic exercise, and can be used exclusively in predicting VO_2_ during steady-state submaximal work. Hence, it will overestimate VO_2_ for peak exercise since contribution from anaerobic metabolism is significant [20], which was confirmed in a previous validation study [24]. However, we were able to validate a model by Uth and colleagues [15] using heart rate ratio (HR_peak_/resting heart rate) as predictor variable for VO_2max_. The Uth model, derived from 46 well-trained men, presented a SEE of 4.5%. This accuracy was considerably lower when validated using HUNT 3 data (18% and 19% SEE in men and women, respectively), which is supported by Esco and colleagues [14] who also observed a substantial reduction in accuracy (SEE of 16.6%), using 109 healthy men to validate the Uth model. This underscores the importance of similar gender, age and physical fitness between the subjects using the model and the subjects used in developing the model to assure best possible accuracy [2,19].

Submaximal VO_2peak_ prediction models are generally outperformed on accuracy by models derived from peak workload[26], which is also the case in this study presenting accuracies (SEE) of 14.1% (R^2^ = 0.55) and 14.4% (R^2^ = 0.56), in men and women, respectively. Moreover, non-exercise based prediction models derived from HUNT 3 fitness data [27] yielded a somewhat better accuracy (12.8% and 14.3% in men and women, respectively) than the present submaximal models, while the present peak models had better accuracy. Previous research reported prediction error in the range 7.3–20.9% [2,16,28–36]. The bench-mark Åstrand-Ryhming nomogram [37] reported accuracy of approximately 10%, which was confirmed when validated by Cink & Thomas [38]. Both Åstrand and Cink observed minor differences between measured and predicted VO_2peak_. However, both used small groups of physically fit college students for their calculations. Validating the Åstrand-Rhyming nomogram using untrained sedentary subjects [39] showed a 26.5% systematic underestimation of VO_2max_. Several peak [13,18,25] and submaximal models [16,29–31,34,35] used cycle ergometer to measure VO_2peak/max_, however, compelling evidence points to a 6–15% lower VO_2peak_ compared to that obtained when running [40–44].

### Cross-validation of VO_2peak_ prediction models

Randomly splitting data into validation and cross-validation samples established good stability throughout all models, suggesting minor shrinkage in accuracy if used on other similar populations. Moreover, data splitting will minimize potential over fitting that might deteriorate the external validity of the models [45].

For the peak performance models, error estimates are fairly stable across subgroups of both age and treadmill velocity. Conversely, in the VO_2peak_ subgroups we observed a trend of systematic under- and overestimation of the predicted values in the high- and low-fit participants, respectively. This is consistent with previous findings [12,46,47].

Similarly for the submaximal models, error estimates are reasonably stable across the treadmill velocity subgroups, whereas across the age subgroups there is a tendency towards under- and overestimating VO_2peak_ in the youngest and oldest, respectively. For the VO_2peak_ subgroups an even greater tendency towards under- and over estimation in the high- and low-fit participants, respectively, is observed compared to the peak performance models. Wier and colleagues [26] argue that the underestimation of the fittest participants is of less importance from a public health perspective, since a high level of fitness is not associated with adverse health outcomes. However, it highlights the necessity of using models derived from aerobically fit subjects to obtain high predictive accuracy and stability for a well-trained population. Such models are previously developed [12,15,18], while models with high predictive accuracy for low-fit populations are scarce. The models inability to accurately identify fitness level in the low-fit subjects represent a potential concern, since low aerobic fitness is associated with increased prevalence of chronic disease as well as a higher mortality risk, e.g. cardiovascular disease and metabolic syndrome [3,4,48]. However, cross-classification accurately predicted approximately 91% of participants, in both sexes, within the nearest quintile of measured VO_2peak_.

There are several factors that might contribute to the systematic over- and underestimation of VO_2peak_, as well as to the attenuation of prediction accuracy. The statistical rationale is that our models are based on linear regression, where the distribution assumptions smooth out extreme observations compared to the grand mean, and may therefore under predict high observations and conversely over predict low observations (regression-to-the-mean phenomenon).

For the submaximal performance models there are additional plausible factors. Genetics account for an additional source of prediction inaccuracy as maximal heart rate is heterogeneous, with significant variations in a population [1]. Based on HUNT 3 fitness data, our group recently reported a standard deviation on measured maximal heart rate of ±14 beats⋅min^-1^ [23]. Consequently, imbedding fraction of maximal/peak heart rate as a separate equation in the model weakens the accuracy of the VO_2peak_ prediction [1]. Furthermore, since the models are based on linear predictions the best trained are underestimated, and could be so because they have a good movement economy, conversely an overestimation of the least fit, attributed poor movement economy. These additional possible explanations are supported by the considerably higher over- and under estimation of VO_2peak_ in the submaximal performance models compared to the peak performance models. Moreover if a person using the prediction equation has a better movement economy than that of the subjects in the HUNT 3 fitness study, he or she will be overestimated using the submaximal model, and conversely underestimated with poor movement economy. The person will have a better or worse aerobic capacity, influenced by movement economy, not by VO_2peak._

### The independent variables influence on VO_2peak_

Calculating standardized β weights, for the models based on peak performance, revealed velocity as the key determinant of VO_2peak_, followed by age and weight, among both sexes. Not surprisingly inclination had the least impact on VO_2peak_, since approximately 87% of the subjects tested on 10% treadmill inclination in the peak performance models.

Likewise for the submaximal models velocity was paramount in determining VO_2peak_ among both sexes. In men importance of succeeding determinants of VO_2peak_ were fraction HR_peak_ (consisting of age and work heart rate), weight and inclination. For women this was altered to inclination, fraction of HR_peak_ and weight. Inclination being more potent in women may be related to a larger diversity in running inclination. Explained variances in the submaximal models were 55% and 56% in men and women, respectively, which yields better predictive capabilities than some (31–51%) [17,31,34], and yet worse than other previous models (60–83%) [2,16,28,29,32,33,35].

### Strengths and Limitations

The large sample size, including both men and women, and wide age range makes this study robust. Our direct test to volitional exhaustion to measure VO_2peak_ by ventilatory gas analysis is preferable compared to indirect estimates when making prediction equations from population studies, since direct measurements display higher correlations as well as lower standard error of estimate [5]. The low participation rate may contribute to bias caused by self-selection. Still, 5633 (45%) of those invited to the present Fitness study from the total HUNT population volunteered for the cardiopulmonary exercise test. Out of these 5633, 1003 candidates withdrew, did not complete the CPET or were excluded for medical reasons, leaving 4631 (37%) completed tests. Some potential candidates declined participation due to long waiting lines caused by limited capacity at test sites. Consequently, it is possible that those who finally partook could be healthier than those who withdrew from testing. However, comparing the Fitness study participants to a healthy sample from the total HUNT population (i.e. free from pulmonary- and cardiovascular diseases, sarcoidosis or cancer) established that there were no considerable differences between the two [3]. However, the consistent overestimation of the least fit candidates associated with the highest health risks is more precarious. This should be taken into account when applying the models.

The models inability to accurately identify fitness level in the low-fit subjects represent a potential concern, since low aerobic fitness is associated with increased prevalence of chronic disease as well as a higher mortality risk, e.g. cardiovascular disease and metabolic syndrome

### Practical implications

In a health care setting the models good ability to detect subjects with low VO_2peak_ is paramount to classify persons in need of physical activity and lifestyle intervention. Cross-classification of participants into quintiles of measured and predicted VO_2peak_ demonstrate the models reasonable ability to classify participants appropriately. More importantly, both the use of peak- and submaximal performance models are considered a generally safe practice on high-risk cardiovascular disease patients [49]. Our models are derived from a large population of both men and women, with a wide heterogeneity in fitness levels as well as covering a large age span (20–90 years). This provides a high degree of applicability for widespread use.

## Conclusions

The VO_2peak_ prediction models presented in this study are inexpensive and uncomplicated to utilize, thus a convenient option for both recreational athletes as well as in health care settings. Judicious and appropriate use of these predictive models will offer valuable information in providing a fairly accurate estimate of peak oxygen uptake, which is beneficial for establishing cardiorespiratory fitness, and with potentially improved risk stratification.
